# Transcriptome profile and immune infiltrated landscape revealed a novel role of γδT cells in mediating pyroptosis in celiac disease

**DOI:** 10.1186/s12967-023-04359-1

**Published:** 2023-07-24

**Authors:** Shuze Chen, Xiuying Liu, Zhi Wang, Dekai Zheng, Ying Wang, Yiling Yan, Xiaojie Peng, Qiujuan Ye, Ye Chen

**Affiliations:** 1 Department of Gastroenterology, Guangdong Provincial Key Laboratory of Gastroenterology, State Key Laboratory of Organ Failure Research, Nanfang Hospital, Southern Medical University, Guangzhou, China; 2 The First School of Clinical Medicine, Southern Medical University, Guangzhou, China; 3 Integrative Clinical Microecology Center, Shenzhen Key Laboratory of Gastrointestinal Microbiota and Disease, Shenzhen Clinical Research Center for Digestive Disease, Shenzhen Technology Research Center of Gut Microbiota Transplantation, Shenzhen Hospital, Southern Medical University, Shenzhen, China; 4 Department of Critical Care Medicine, Nanfang Hospital, Southern Medical University, Guangzhou, China

**Keywords:** Celiac disease, γδT cells, Pyroptosis, Interferon-γ, Gluten, Immunity

## Abstract

**Background:**

Celiac disease (CeD) is a primary malabsorption syndrome with no specific therapy, which greatly affects the quality of life. Since the pathogenesis of CeD remains riddled, based on multiple transcriptome profiles, this study aimed to establish an immune interaction network and elucidated new mechanisms involved in the pathogenesis of CeD, providing potentially new evidence for the diagnosis and treatment of CeD.

**Methods:**

Three microarray and three RNA sequencing datasets of human duodenal tissue with or without CeD were included in Gene Expression Omnibus and respectively merged into derivation and validation cohorts. Differential expression gene and functional enrichment analysis were developed, then pyroptosis enrichment score (PES) model was established to quantify pyroptosis levels. Immune infiltration and co-expression network were constructed based on Xcell database. Protein–protein interaction and weighted gene co-expression network analysis were determined to identify pyroptosis relative hub genes, whose predictive efficiency were tested using a least absolute shrinkage and selection operator (LASSO) regression model. CeD animal and in vitro cell line models were established to verify the occurrence of pyroptosis and molecules expression employing immunofluorescence, western blotting, cell counting kit-8 assay and enzyme-linked immunosorbent assay. Analysis of single-cell RNAseq (scRNAseq) was performed using “Seurat” R package.

**Results:**

Differentially expressed genes (DEGs) (137) were identified in derivation cohort whose function was mainly enriched in interferon response and suppression of metabolism. Since an enrichment of pyroptosis pathway in CeD was unexpectedly discovered, a PES model with high efficiency was constructed and verified with two external databases, which confirmed that pyroptosis was significantly upregulated in CeD epithelia. γδT cells exhibited high expression of IFN-γ were the most relevant cells associated with pyroptosis and occupied a greater weight in the LASSO predictive model of CeD. An accumulation of GSDMD expressed in epithelia was identified using scRNAseq, while animal model and in vitro experiments confirmed that epithelium cells were induced to become “pre-pyroptotic” status via IFN-γ/IRF1/GSDMD axis. Furthermore, gluten intake triggered pyroptosis via caspase-1/GSDMD/IL-1β pathway.

**Conclusion:**

Our study demonstrated that pyroptosis was involved in the pathogenesis of CeD, and elucidated the novel role of γδT cells in mediating epithelial cell pyroptosis.

**Supplementary Information:**

The online version contains supplementary material available at 10.1186/s12967-023-04359-1.

## Background

Celiac disease (CeD) is a human leukocyte antigen-linked autoimmune-mediated enteropathy, which is triggered by the ingestion of gluten in genetically predisposed individuals. With a worldwide prevalence of 1%, this disorder presents with broad clinical manifestations, including intestinal and extra-intestinal symptoms, and is therefore a considerable global public health concern [1]. Currently, the only available therapy for CeD patients is a life-long exclusion of gluten from the diet, but it is associated with several possible challenges, including inferior quality of life, inadequate response, possible negative effects, and a heavy economic and societal burden [2]. Therefore, more novel therapeutic approaches are urgently needed.

Previous studies on the pathogenesis of CeD have suggested that CD4^+^ T cells play an important role in recognising deamidated gluten peptides by binding to HLA-DQ molecules HLA-DQ2.5, HLA-DQ2.2, and HLA-DQ8 in antigen-presenting cells. Then, gluten-specific CD4^+^ T cells support cytotoxic CD8^+^ T cells in expressing high levels of activated NK receptors to kill enterocytes and inducing B cells to produce gliadin-associated antibodies [3, 4]. Moreover, it has been shown that the percentage of γδTCR^+^IELs was increased in duodenal biopsies from CeD patients [5], which might play a proinflammatory role in the process of gut damage [6]. A chronic site-specific inflammation triggered by gluten permanently reconfigures the tissue-resident γδTCR^+^ IEL compartment in patients with CeD, accompanied by the expansion of gluten-sensitive, interferon-γ-producing γδTCR^+^ IEL [7]. In addition, there are many other immune cells in the gut that are involved in the development of CeD, and more detailed mechanisms of these cells need to be explored further. Therefore, this study involved a comprehensive analysis by integrating multiple‐microarray and transcriptome sequence analysis, then constructed an immune atlas and the pathogenesis network of CeD and attempted to provide potential targets for the clinical diagnosis and treatment of this disorder.

## Methods

### Establishment of celiac disease (CeD) animal model

Female pregnant Wistar rats, purchased from the Laboratory Animal Center of Southern Medical University (Guangzhou, China), were maintained in specific pathogen free conditions and had free access to food and water. The animal handling protocols were approved by The Institutional Animal Care and Use Committee of Southern Medical University (Guangzhou, China).

Shortly after spontaneous birth, rat pups were randomly distributed into two groups. In detail: (1) “Control group” (artificially reared without treatment) (n = 6); (2) “IFN-γ/gliadin group” (sensitized by intraperitoneal injection of 1000U interferon-γ (IFN-γ) and treated with Pepsin-trypsin gliadin 50 μg/day for 10 days) (n = 6). All newborn pups were fed on a rat milk formula every 4 h until 10 days. A dose of 1000U IFN-γ was derived from recombinant rat IFN-γ (PeproTech, China). Pepsin-trypsin gliadin (PTG, gliadin from wheat gluten, Sigma-Aldrich) was obtained following a previously published procedure [8]. After 10 days, the anesthetized animals were sacrificed for further studies.

### Cell cultures and treatments

Human colon adenocarcinoma-derived cells (Caco-2) obtained from the ATCC (Rockville, USA) were cultured in DMEM, supplemented with 10% fetal bovine serum, 100 units penicillin–streptomycin/mL, and 1 mmol/L glutamine (all these products are Gibco Invitrogen, Milan, Italy). Cells were maintained in a humidified atmosphere (95%) of air and 5% CO2 at 37 °C.

To knock GSDMD down, GSDMD target siRNA (GTGTGTCAACCTGTCTATCAA) or control nonspecific siRNA (Ribobio, Guangzhou, China) were transfected into Caco-2 cells with Advanced DNA RNA Transfection Reagent (ZETA LIFE, USA) following the manufacturer’s protocol. After 24 h, the cells were treated with or without IFN-γ (Peprotech, USA) in different concentrations, and the cells and culture supernatants were collected after 24 h.

PTG were dissolved in 100 μl of DMSO, and then diluted it in 99.9 ml of DMEM to prepare 200 μg/ml of PTG medium solution. To evaluate the effect of IFN-γ and PTG, Caco-2 cells were divided into 4 groups and incubated with 50 ng/ml IFN-γ only, 200 μg/ml PTG only, both 50 ng/ml IFN-γ and 200 μg/ml PTG, and neither of them, respectively. After 24 h, the cells and media were harvested for further studies.

### Cell counting kit-8 (CCK-8) assay

Caco-2 cells were mixed with 10 µl CCK-8 solution (Fude, Hangzhou, China) and incubated at 37 °C for 2 h, and their absorbances at 450 nm were analyzed via the microplate reader (Molecular Devices, USA) to determine the cell viability.

### Enzyme-linked immunosorbent assay (ELISA)

ELISA kit (JSBOSSEN, BS-E3947H2, China) was used to measure the production of IL-1β in culture supernatants following the manufacturer’s protocol. Briefly, testing samples and standards were implanted in 96-well plates and incubated with the conjugate reagents for 1.5 h. Then the TMB solution was added to each well, and the samples were incubated at 37 °C for another 15 min. Lastly, the stop solution was added to terminate the reaction, and the absorbance at 450 nm was measured using the microplate reader (Molecular Devices, USA).

### Immunofluorescence

According to the standard protocols, paraffin-embedded duodenal tissue sections (4 μm) underwent an appropriate heat-induced antigen retrieval. Samples were blocked for 1 h, and incubated overnight at 4 °C with FITC-anti-rat-CD3 and PE-anti-rat-TCRγδ (Biolegend, San Diego, CA, USA). The next day samples were washed and stained with DAPI for 10 min, and imaged via fluorescent microscopy (Olympus, Japan).

### Western blotting

Protein extracts were obtained from cells and rat duodenal tissue samples employing total lysis buffer (Beyotime, Shanghai, China) supplemented with protease and phosphatase inhibitors (Fudebio, Hangzhou, China). After homogenization and centrifugation at 14,000 rpm for 15 min at 4 °C, concentration of protein supernatant was measured using a standard Bradford assay (ThermoFisher Scientific, Rockford, USA). The samples were resolved using SDS-PAGE, then transferred onto PVDF membranes (EMD Millipore, Billerica, Massachusetts, USA), and blocked with 5% skim milk for 120 min. The blocked membranes were incubated at 4 °C overnight with corresponding primary antibodies targeting GSDMD full length and cleaved GSDMD (C Teminal) (Abcam, USA), Caspase-1 (Proteintech, Wuhan, China), IL-1β (Abcam, USA), β-Tublin (Abmart, Shanghai, China), Cleaved Caspase-1 (CST, USA)) followed by secondary antibodies (Proteintech, Wuhan, China) at room temperature for 120 min. Lastly, the detection was performed using the enhanced chemiluminescence detection kit (Yeasen, Shanghai, China).

### Quantitative reverse transcription PCR (qRT-PCR)

Total RNA of cell were extracted by TRIzol RNA extraction agent (Thermo Fisher Scientific, USA) according to manufacturer manual. RNA concentration were measure by absorbance at 260 nm, then reverse transcribed via reverse transcriptase kit (Vazyme Biotech Co.,Ltd, China) followed by real-time PCR amplification by use of SYBR Green Premix ExTaq (Vazyme Biotech Co., Ltd, China). Data were interpreted using the 2^−ΔΔCt^ method, with GAPDH serving as the reference gene for normalization. The primer of GSDMD were: GTGTGTCAACCTGTCTATCAAGG (forward strand) and CATGGCATCGTAGAAGTGGAAG (reserved strand). The primer of GAPDH were: GGAGCGAGATCCCTCCAAAAT (forward strand) and GGCTGTTGTCATACTTCTCATGG (reserved strand).

### Data acquisition

In the Gene Expression Omnibus database (GEO https://www.ncbi.nlm.nih.gov/geo/), we selected several datasets related to CeD for analysis. Among them, three microarray datasets, GSE72625 [9], GSE112102 [10], GSE164883 [11] were based on the GPL10558 platform and were merged into the derivation cohort, including 48 CeD patients and 51 healthy control (HC)’s duodenal tissues. Validation cohort was developed using three transcriptome high-throughput sequencing datasets (GSE131705 [12], GSE134900 [13] and GSE146190 [14]), consisting of duodenal tissue of 90 CeD patients and 70 HCs. Dataset GSE123649 [7] contained samples of TCRγδ^+^ intraepithelial lymphocytes in the duodenum of 18 active CeD patients and 8 CeD patients receiving gluten-free diet (GFD) and 8 HCs. Dataset GSE106260 included duodenal epithelial cell samples of 4 CeD patients and 4 HCs [15]. External datasets associated with pyroptosis, including GSE125625 [16] and GSE191015 [17], were used to further verify reliability of pyroptosis enrichment score. Single cells RNA sequence dataset GSE195780 [18] (including epithelial cells and lamina propria CD45^+^ immune cells in CeD duodenal tissues), were included for further analysis. The dataset GSE145358 [19] contained 15 duodenal tissues from CeD patients received GFD and 15 duodenal tissues from GFD patient accepted gluten challenge (10 weeks, 4 g of gluten daily).

### Data processing and identification of differentially expressed genes

Datasets were merged and removed the batch effect with the R package “sva” [20], and then visualized in a boxplot and examined by principal component analysis (PCA). Differential expression genes (DEGs) analysis of microarray data were performed using the R package “limma”[21]. In high-throughput sequencing datasets, the “DEseq2” R package [22] was used for detecting DEGs. An adjust *P*-value < 0.05 and log Fold Change (logFC) > 2 were considered statistically significant.

### GO, KEGG and reactome enrichment analysis

By using R packages “clusterProfiler” [23], the DEGs biological function analysis was carried out based on Gene Ontology (GO) [24] (http://geneontology.org/) and Kyoto Encyclopedia of Genes and Genomes (KEGG) pathway database [25] (https://www.genome.jp/kegg/pathway.html). An adjusted *P* value < 0.05 (via the Benjamini–Hochberg method) was set as the cut-off criterion. The enrichment results were visualized by R packages “enrichplot” (https://github.com/YuLab-SMU/enrichplot) and “ggplot2” (https://ggplot2.tidyverse.org). Reactome pathway analysis was developed according to the “Reactome Pathway Database” (https://reactome.org/), and the results were visualized via the same method.

### Construction of protein–protein interaction (PPI) network and recognition of hub genes

The Search Tool for the Retrieval of Interacting Genes (STRING) online database (http://string-db.org; version11.0) was used to construct PPI networks for the DEGs, and an interaction score > 0.4 was regarded as statistically significant. Subsequently, the molecular interaction network was visualized using Cytoscape software (https://cytoscape.org/). Furthermore, we used the MCODE plugin app (https://sourceforge.net/projects/mcode/) within Cytoscape to identify hub genes and calculate the degrees of interaction between DEGs to visualize the ranking.

### Single sample gene set enrichment analysis (ssGSEA) and definition of pyroptosis enrichment score (PES)

To quantitatively describe the pyroptosis degree of each sample, the parameter PES was constructed using the ssGSEA algorithm based on the R package “GSVA” [26]). Briefly, after identifying all differentially expressed pyroptosis genes between CeD and HC samples, the “pro-pyroptotic” genes and the “anti-pyroptotic” genes were defined as upregulated and downregulated genes in CeD samples, respectively. The positive pyroptosis pathway components were calculated by the ssGSEA scores of upregulated PRGs, and the negative pathway components were calculated by the ssGSEA scores of downregulated PRGs. The PES was calculated as normalized positive pyroptosis pathway components minus normalized negative pyroptosis pathway components. In addition, the enrichment score of “Response to IFN-γ” pathway was also calculated empolying ssGSEA algorithm based on the GO database (GO:0034341).

### Immune cell infiltration estimation and co-expression network analysis

By applying *xCell* [27] to the microarray data, the estimated proportion of immune and stromal cell types can be obtained for each duodenal sample in the derivation and validation cohorts. The cut-off value for the cell analysis was *P* < 0.05. Cell types were categorized into lymphoid, myeloid, stromal, epithelial and stem cells. Furthermore, to elucidate the interactions between immune cells and pathways and explore critical immune cells in CeD pathogenesis, we identified co-expression patterns based on Spearman correlation analysis.

### Weight gene correlation network analysis (WGCNA)

WGCNA were performed in the gene expression profiles from the derivation cohort, while the characteristics matadata were consisted of PES, infiltration of γδT cells level and IFN-γ response score. At first, the *hclust* function was used for hierarchical clustering analysis. Then, the soft thresholding power value was screened during module construction using the *pickSoftThreshold function.* Candidate power (1 to 30) was used to test the average connectivity degrees of different modules and their independence. A suitable power value was selected if the independence degree was > 0.8. The “WGCNA” R package [28] was used to construct co-expression networks (modules). The minimum module size was set to 12, and each module was labelled with a different color. Finally, the gene module that most relevant to γδT cells infiltration, IFN-γ response and pyroptosis was extracted to identifying hub genes.

### Construction of the least absolute shrinkage and selection operator (LASSO) regression model

The LASSO regression model was established to further verify the diagnostic or predictive effectiveness of PES, pyroptosis-associated hub genes or γδT cells infiltration in CeD. In this model, variables from the derivation cohort were used to construct the LASSO model by the R package “glmnet” (https://CRAN.R-project.org/package=glmnet). The reliability of the LASSO model was evaluated by Receiver Operating Characteristic (ROC) analysis using the R package “ROCR”. The repeatability of the variables included in the LASSO model was verified in the validation cohort using the Ridge regression model.

### Gene set enrichment analysis (GSEA)

GSEA is a calculation method used to determine whether predefined genomes between two groups exhibit significant differences. Therefore, we used the GSEA software (https://www.gsea-msigdb.org/gsea/index.jsp) to screen GO terms and KEGG pathways that may be related to γδT cells in duodenal tissues in the derivation cohort. The GSEA analysis was performed according to default parameters. *P* value < 0.05 was considered significant.

### Single-cell sequencing analysis (scRNAseq)

In this study, one sample “GSM5850285RCDII-2” from dataset GSE195780 were selected for scRNAseq analysis. Seurat package v4.0.3 (https://satijalab.org/seurat/articles/pbmc3k_tutorial.html) was used in R version 4.1.3. The Seurat object was constructed under the criteria that retain only genes expressed in at least 3 cells and cells with at least 200 detected genes. After calculating mitochondrial gene proportion, a second quality control was carried out to remove cells with unique gene cell counts fewer than 200 or more than 5000, while mitochondrial gene percentage was greater than 15%. Next, the data was transformed through log-normalization and linear scaling. Two thousand most highly variable features were selected for PCA. The top 18 PCA dimensions were used for graph-based clustering. With a resolution of 0.9, the clusters were dimensionally reduced and visualized employing a Uniform Manifold Approximation and Projection (UMAP) algorithm.

### Statistical analysis

The SPSS 26.0 software (SPSS, Chicago, IL, USA) was used for statistical analysis. Results were described as mean ± standard deviation (SD) or median contains upper and lower quartiles. The Kolmogorov–Smirnov test was used to assess whether the data accorded with normal distribution. Statistical comparison was performed using the Student *t*-test or one-way analysis of variance (ANOVA) for normal distribution variables, and Kruskal–Wallis test and the Mann–Whitney *U* test for Non-normal distribution variables. *P* < 0.05 indicated a statistically significant difference.

## Results

### Differences in transcriptome profiles between human duodenal tissue with or without CeD

**Fig. 1:**
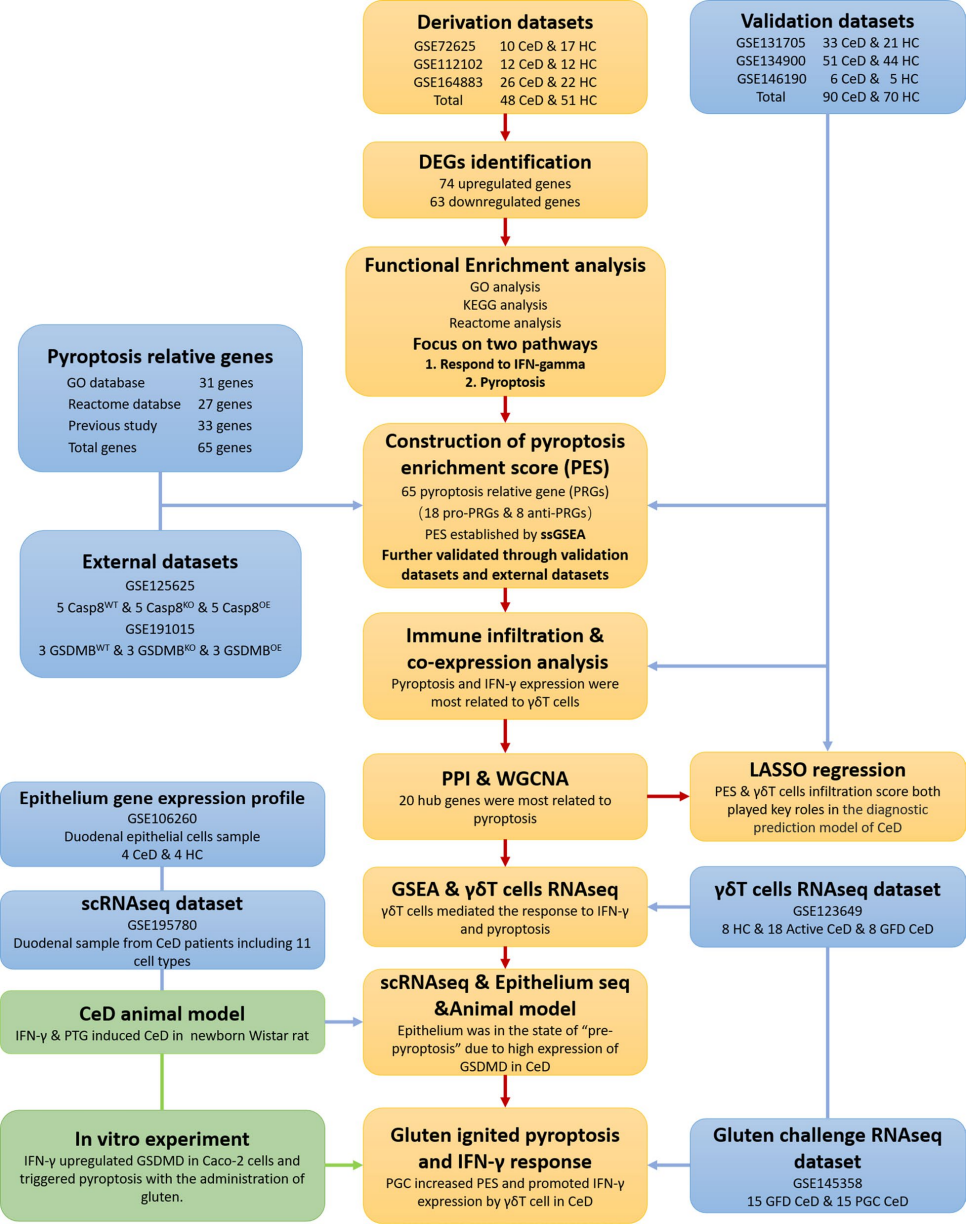
Workflow of the study and data preprocessing of the derivation and validation cohorts. The box in orange represented the mainstream logic of the study, while the blue box represented the validated data and the green box represented experiments

The workflow of this study was demonstrated in Fig. 1. To explore the gene transcriptome profiles of duodenal tissue from patients with CeD or healthy control (HC), the derivation and validation cohorts were developed as described in the methodology section (Additional file 1: Fig. S1A, B).

**Fig. 2:**
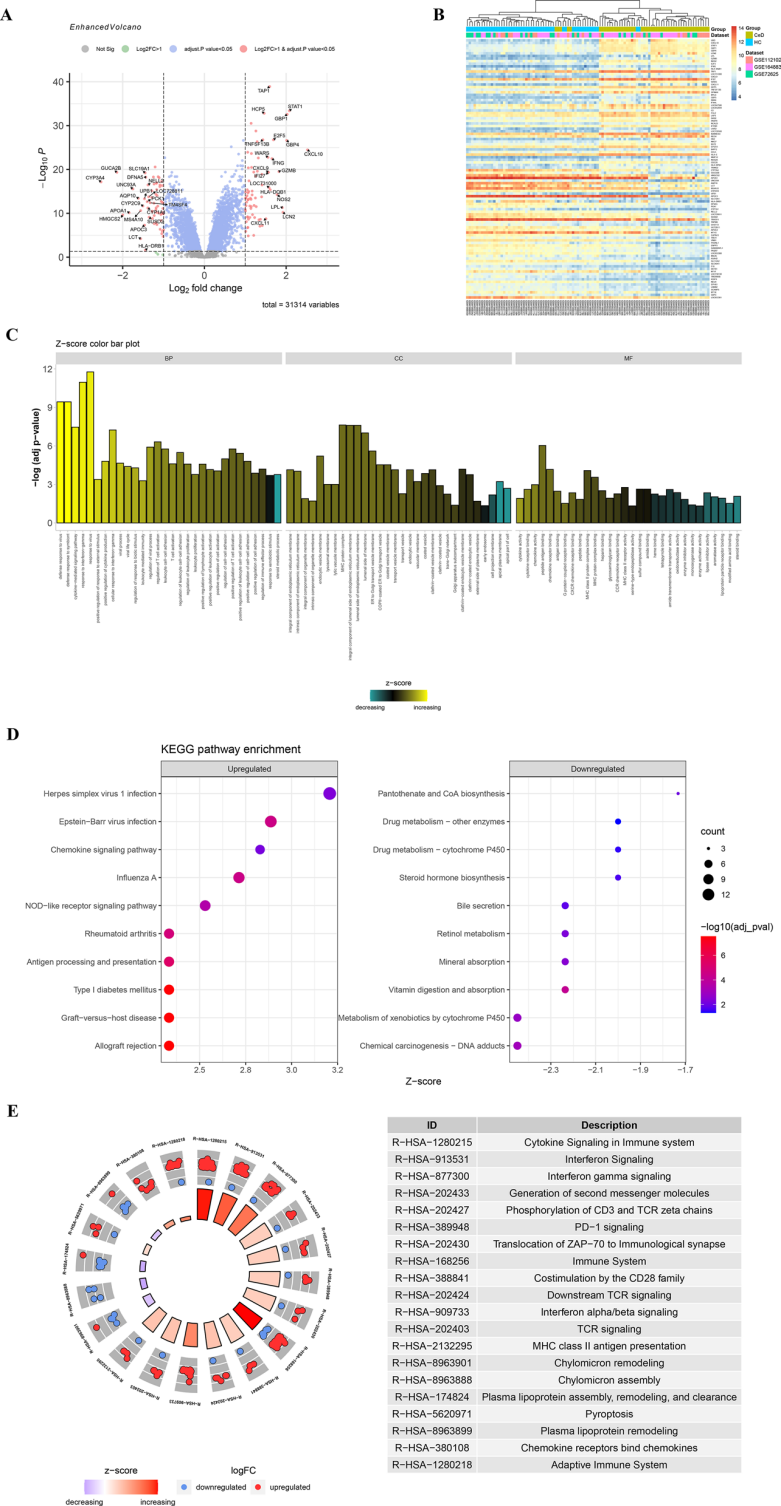
Differentially expressed genes (DEGs) analysis and gene functional enrichment analysis between CeD and HC tissues. **A** Volcano map of the top 20 upregulated genes and top 20 downregulated genes. **B** Heatmap of a total of 137 differentially expressed genes with unsupervised clustering. **C** The bar diagram showed Gene Ontology enrichment analysis of DEGs based on Z-score. “BP” referred to “Biological Process,” “CC” referred to “Cellular Component” and “MF” referred to “Molecular Function.” **D** The bubble plot exhibited KEGG pathway analysis of DEGs with 10 most activated and 10 most inhibited pathways. **E** The trigram array plot demonstrated the Reactome pathway analysis of DEGs and the top 20 enrichment pathways

According to the cut-off criteria (fold change > 2 and adjust *P* value < 0.05), a total of 137 differentially expressed genes (DEGs) (74 upregulated and 63 downregulated genes) were identified between CeD and HC duodenal tissues in derivation cohort (Fig. 2A, Additional file 4: Table S1). An unsupervised clustering based on DEGs expression profiles illustrated that the classification of samples was generally consistent with their groups (Fig. 2B), revealing that patients in CeD groups showed a different transcriptome profile in their duodenal tissue compared with HC counterpart.

### Functional annotations of DEGs reveals the involvement of pyroptosis and IFN-γ-mediated response in the pathogenesis of CeD

Gene Ontology (GO), Kyoto Encyclopedia of Genes and Genomes (KEGG) and Reactome analyses were performed to annotate the DEGs function. Unsurprisingly, several pathways associated with the response to IFN-γ were significantly activated in the CeD group, while metabolism-related pathways, including lipid metabolism and mineral absorption, were downregulated in CeD according to the GO and KEGG analyses (Fig. 2C, D, Additional file 5: Table S2, S3).

Similar results were also reproduced in the Reactome pathway analysis (Fig. 2E, Additional file 5: Table S4). Interestingly, one result indicated that the pyroptosis process was activated in CeD tissue, which had never been reported in previous studies. Pyroptosis, also known as inflammatory necrosis, is a kind of programmed cell death, which is manifested by the continuous expansion of cells and rupturing of cell membranes, resulting in the release of cell contents, such as IL-1β, leading to strong inflammatory reactions [29]. Since the atrophy and apoptosis of epithelial cells are important pathophysiological changes in CeD, we have inferred that pyroptosis may also be involved in the pathogenesis of CeD.

### Construction and verification of a computational model that reveals the upregulation of pyroptosis in CeD

**Fig. 3:**
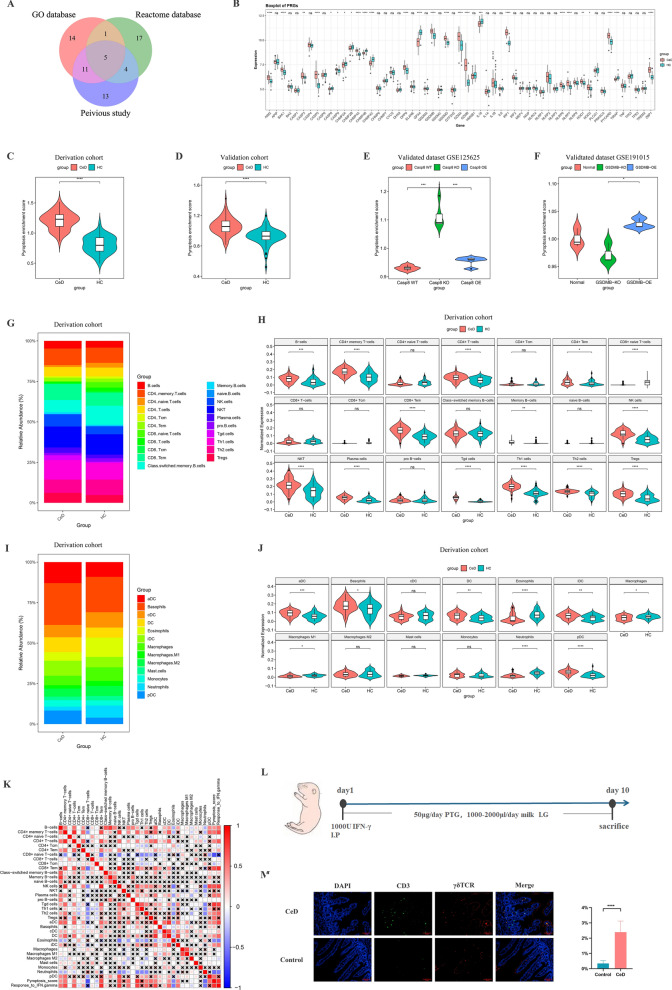
Computational modeling of the pyroptosis enrichment score (PES) and construction of immune infiltration co-expression network. **A** The venn diagram of pyroptosis related genes identified in three databases (GO database, Reactome database, and previous studies). **B** The boxplot showed the comparison of pyroptosis related genes between CeD and HC tissues in the derivation cohort. **C** The boxplot showed the differences in the PES between CeD and HC tissues in the derivation cohort. **D** The boxplot showed the differences in the PES between CeD and HC tissues in the validation cohort. **E** The boxplot showed the difference comparison of PES among three groups in dataset GSE125625. **F** The boxplot showed the difference comparison of PES among three groups from dataset GES191015. **G** The stacking histogram exhibited the infiltration proportion of different lymphoid cell types between CeD and HC in the derivation cohort. **H** Difference comparison of the infiltration score of various lymphoid cell types between CeD and HC in the derivation cohort. **I** The stacking histogram exhibited the infiltration proportion of different myeloid cell types between CeD and HC in the derivation cohort. **J** Difference comparison of the infiltrated score of various myeloid cell types between CeD and HC in the derivation cohort. **K** The correlation heatmap reflected co-expression patterns among immune cell infiltration score, PES, and response to IFN-γ pathway in the derivation cohort. **L** Establishment of the celiac disease rat model. **M** Representative immunofluorescence images showed the distribution of γδT cells in duodenal tissues of CeD rats and the statistical analysis of γδT cells infiltration degree quantified by the proportion of CD3^+^ γδTCR^+^ IELs to total IECs

To further determine the role of pyroptosis in CeD and quantitatively describe the pyroptosis level, we constructed a scoring model based on ssGSEA algorithm. Firstly, a total of 65 pyroptosis-associated genes (PAGs) were identified from the Reactome pathway analysis database (27 genes), the GO (31 genes), and previous reports (34 genes) [30, 31] (Fig. 3A, Additional file 6: Table S5). Next, DEGs analysis of these PAGs was performed in derivation cohort, which identified 18 upregulated genes and 8 downregulated genes in CeD group (Fig. 3B, Additional file 7: Table S6).

Here, we innovatively established a parameter “pyroptosis enrichment score (PES)” in the derivation cohort (Fig. 3C). The reliability of PES was synchronously verified in the validation cohort and two datasets from studies associated with pyroptosis. It was shown that the PES was significantly higher in CeD patients in both derivation and validation cohorts (Fig. 3C, D). In dataset GSE125625, the PES was notably upregulated in Casp8^KO^ group (small intestinal tissue from pyroptotic suppressed gene Casp8 knockout mouse) compared with Casp8 wildtype and Casp8^OE^ (with Casp8 overexpression) (Fig. 3E). Furthermore, a significant difference in PES was observed between GSDMB^KO^ group (with pyroptosis promoted gene GSDMB knockout) and GSDMB^OE^ group (with GSDMB overexpression) in dataset GSE191015 (Fig. 3F). These results confirmed the reliability of PES and indicated that pyroptosis participated in the progress of CeD. In addition, due to the significantly activated response to IFN-γ pathway in the CeD group, we constructed a new pathway score “Response to IFN-γ” in the same way according to GO database (GO:0034341) (Additional file 1: Fig. S1C–E).

### Immune infiltration and correlation network analysis reveals the association of γδT cells with pyroptosis and IFN-γ response

Previous studies suggested that pyroptosis or IFN-γ response was mainly mediated by immune cells in the gastrointestinal tract [32]. Therefore, to identify the immune cell types that are potentially involved in CeD, we performed an Xcell-based algorithm to generate cell type enrichment scores in both derivation and validation cohorts. The infiltration degree of various immune cells was different between CeD and HC tissues (Additional file 1: Fig. S1F). A total of 23 significantly different immune cells between the two groups were determined, including 14 lymphoid cells (B, CD4^+^ T, CD4^+^ memory T, CD4^+^ Tem, CD8^+^ naïve T, CD8^+^ Tem, memory B, plasma, NK, NKT, Tgd, Th1, Th2 and Treg cells) (Fig. 3G, H), 9 myeloid cells (basophiles, eosinophiles, neutrophils, dendritic cells (DCs), activated DCs, immature DCs, macrophages, macrophages M1, and plasmacytoid DCs (Fig. 3I, J). Other types of cells such as stromal, epithelial and stem cells also showed obvious differences between the two groups (Additional file 1: Fig. S1F).

For instance, a correlation network analysis among the infiltration of immune cells and the two pathways above was performed to further explore which type of immune cells was mostly associated with pyroptosis. Nearly all kinds of T cells significantly correlated with IFN-γ pathway (Fig. 3K). To our surprise, the γδT cells (Tgd cells), were most related to pyroptosis and IFN-γ response (Fig. 3K). Similar results were also obtained for the validation cohort (Additional file 1: Fig. S1G, H), which expounded a potential relationship between γδT cells and pyroptosis during the pathogenesis of CeD. Moreover, the increasing infiltration of CD3^+^γδTCR^+^ intraepithelial lymphocyte cells (IELs) were also found in CeD animal model (Fig. 3L, M), which further strengthened the important role of γδT cells in the disease.

### Identification of pyroptosis-related gene modules via comprehensive analysis of PPI network and WGCNA

**Fig. 4:**
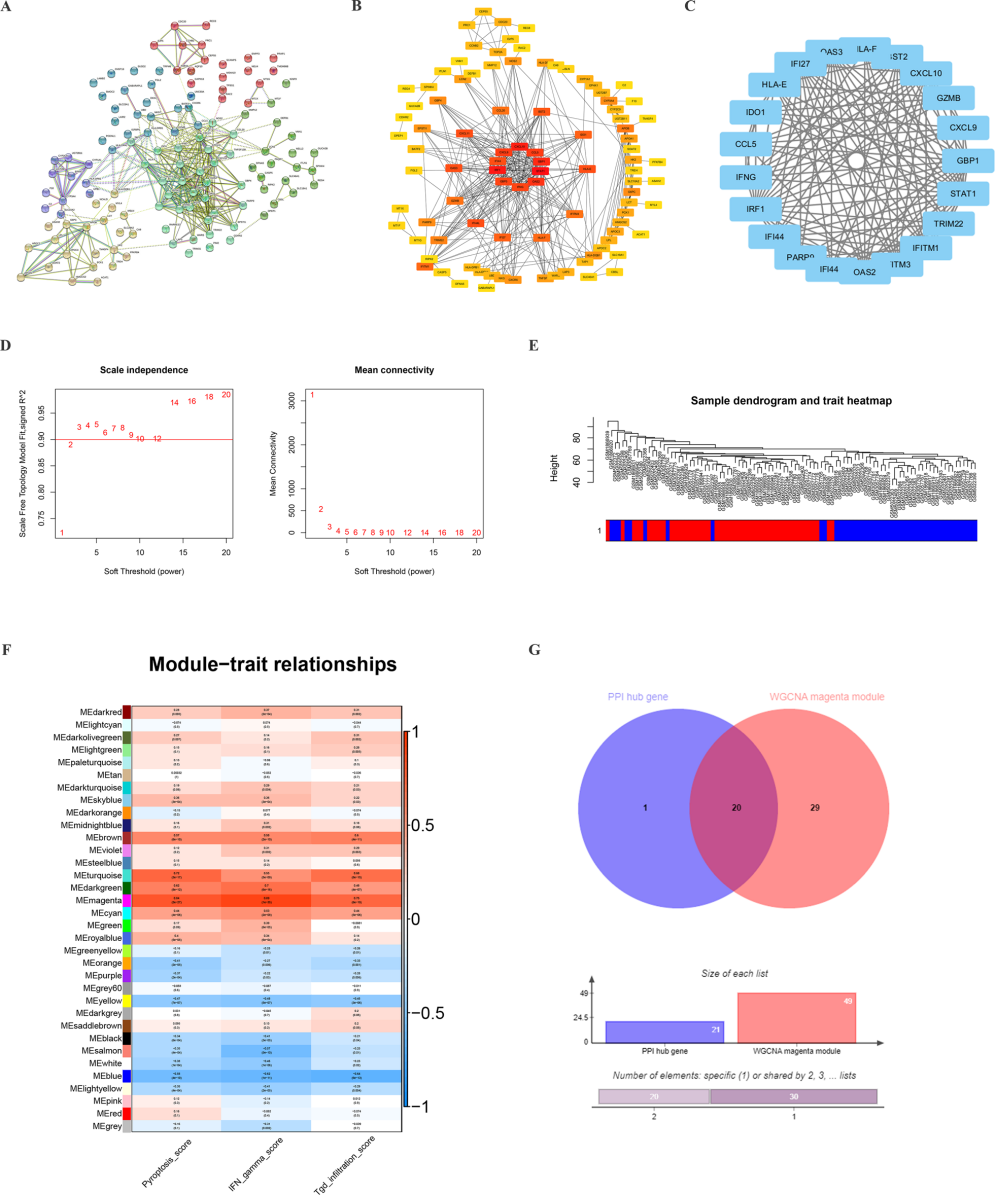
Identification of gene modules related to pyroptosis via comprehensive analysis of PPI network and WGCNA. **A** PPI network was constructed using the STRING database. **B** PPI network was visualized using Cytoscape software and the relevance level were ranked in different colors. **C** Twenty-one hub genes and their connections were identified via the MCODE algorithm. **D** Determination of WGCNA soft threshold. **E** Gene clustering tree (dendrogram) was obtained by hierarchical clustering of adjacency-based dissimilarity. **F** The correlation heatmap of gene modules and phenotypes. **G** The venn diagram showed the genes shared by PPI hub genes and the WGCNA magenta module

A PPI network of DEGs (derivation cohort) was constituted by the STRING database and visualised using Cytoscape tool. Altogether, 125 nodes and 371 edges were established in the PPI net (Fig. 4A, B). Meanwhile, 21 hub genes were recognised from the most significant module, which was constructed by MCODE plug (Fig. 4C).

To further explore which gene or gene module mediates the relationship between γδT cells and pyroptosis, we performed WGCNA using expression profiles of the derivation cohort. Thirty-four modules were constructed when the soft-threshold power was defined as 12 (Fig. 4D, E), then the relationship networks among all the modules and these pathways were established. Interestingly, one of the modules (magenta modules) was strongly related to the pyroptosis pathway, IFN-γ response, and γδT cell infiltration score simultaneously (Fig. 4F). This magenta module contained 49 genes, covering 20 hub genes that we had previously identified (except for IDO1) (Fig. 4G). These results suggested that these 20 hub genes might play a significant role in the pyroptosis pathway.

### Least absolute shrinkage and selection operator (LASSO) regression model demonstrates essential roles of γδT cells and pyroptosis in CeD pathogenesis

**Fig. 5:**
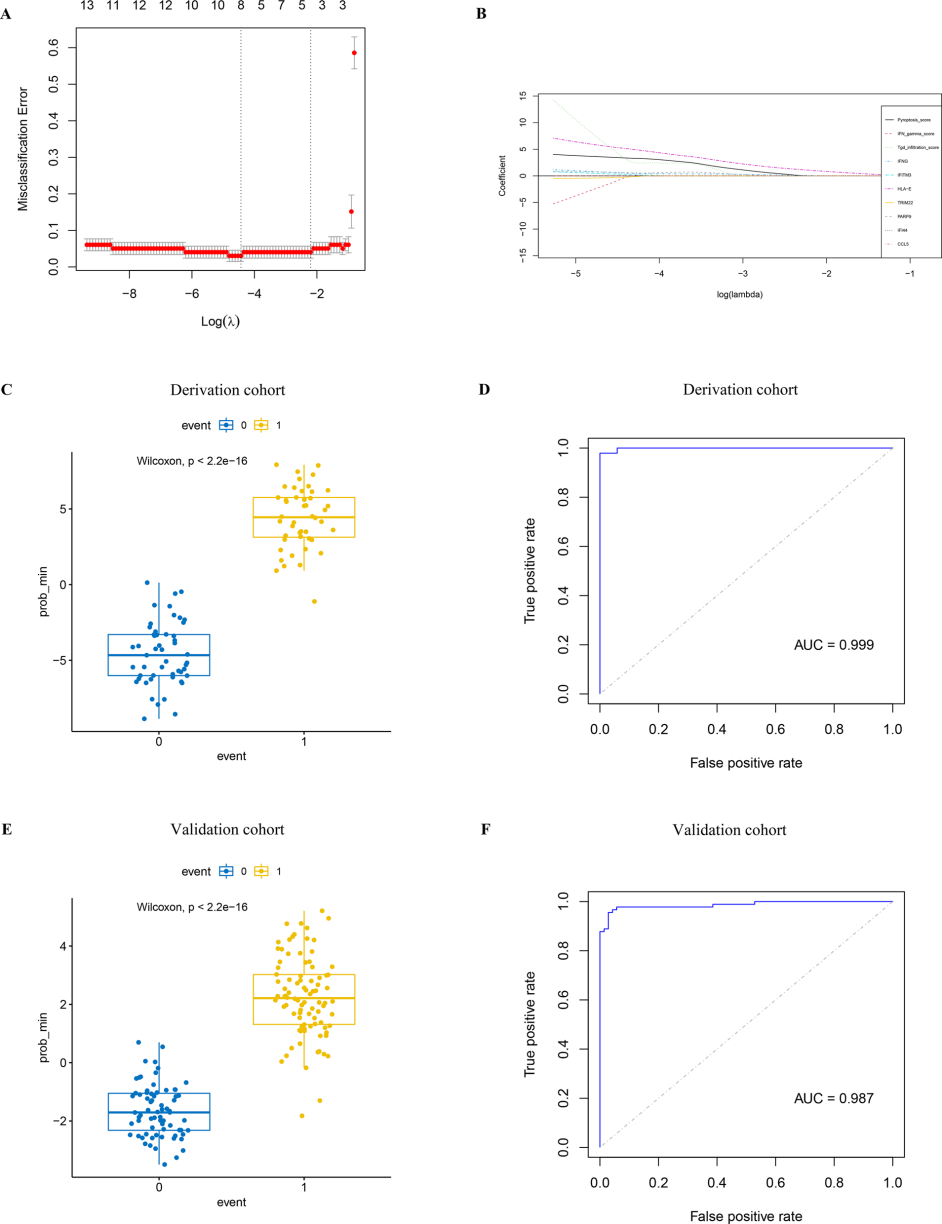
The LASSO regression model for predicting CeD. **A** Screening for lambda value in the LASSO model. **B** Model included variables under minimal lambda value. **C** Prediction efficiency of the LASSO model in the derivation cohort. **D** ROC curves analysis of the prediction efficiency in the derivation cohort via calculating AUC. **E** Prediction efficiency of the LASSO model in the validation cohort. **F** ROC curves analysis of the prediction efficiency in the validation cohort via calculating AUC

Twenty hub genes most related to pyroptosis, PES, score of response to IFN-γ pathway, and γδT cells infiltration abundance were included to construct a LASSO regression model to explore whether these indicators had potential predictive value for the incidence of CeD. Finally, the LASSO model included 10 variables. While the value of lambda.min was 0.0119 [Log(lambda) = − 4.431], the standardised regression coefficients of each independent variable was stable (Fig. 5A). Among them, the γδT cells infiltration score that played the most significant role in the prediction of CeD, followed by the PES (Fig. 5B), which further illustrated that both were decisive in the pathogenesis of CeD. Furthermore, this model showed considerable predictive effectiveness via difference analysis of effective rate (Fig. 5C) and AUC value of ROC analysis of the derivation cohort (AUC = 0.999) (Fig. 5D) and validation cohort (AUC = 0.987) (Additional file 2: Fig. S2A, B and Fig. 5E, F). These results again stressed the importance of pyroptosis and relative hub genes and pathway.

### Identification of the intrinsic relationship among pyroptosis, IFN-γ response, and γδT cells using single-cell GSEA analysis and γδT single-cell bulk transcriptome analysis

**Fig. 6:**
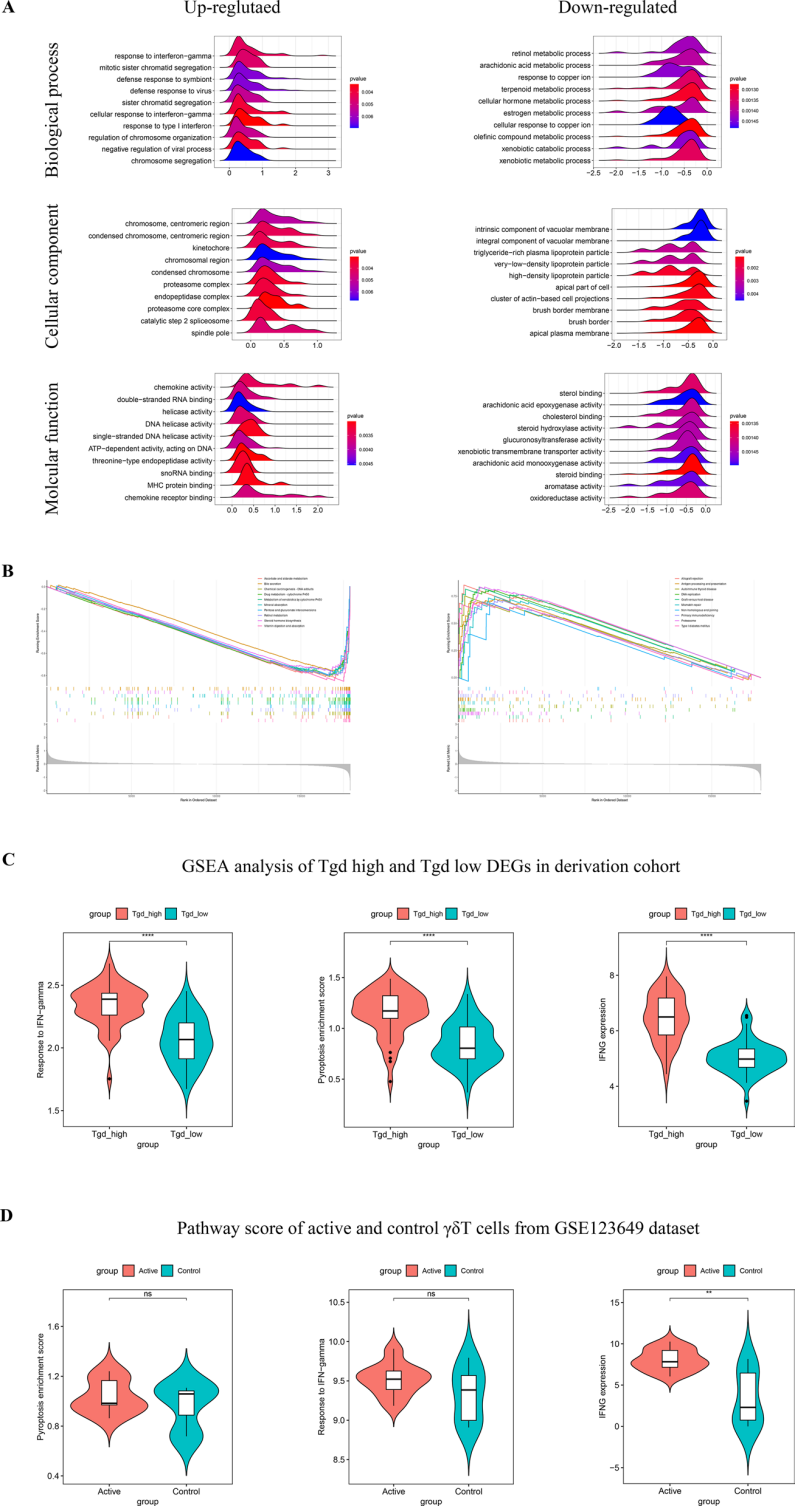
The functional changes mediated by γδT cells and the functional alteration of γδT cells itself during CeD pathogenesis. **A** GSEA analysis between high and low γδT cells infiltration samples in the derivation cohort from biological process, cellular component and molecular function aspects. **B** GSEA analysis of KEGG pathway between high and low γδT cells infiltration samples in the derivation cohort. **C** The comparison of PES, response to IFN-γ pathway and IFNG expression level between high and low γδT cells infiltration samples in the derivation cohort. **D** The comparisonof PES, response to IFN-γ pathway and IFNG expression level between γδT cells from CeD patients and HCs

A correlation among pyroptosis, IFN-γ response, and γδT cells infiltration was demonstrated. However, whether pyroptosis occurred in γδT cells or just induced by γδT cells remained unclear. Thereby, we performed a single-cell GSEA analysis of the derivation cohort to study the difference in biological function enrichment degree between high and low γδT cell infiltration samples. GO analysis showed that upregulated pathways were still enriched in IFN-γ induced cellular response and transcriptional regulation (Fig. 6A). Additionally, the KEGG analysis generated similar results (Fig. 6B). PES, response to IFN-γ pathway, and IFN-γ expression were all elevated in high γδT cell infiltration samples, which further confirmed that those two pathways were associated with γδT cells (Fig. 6C) (see Additional file 7: Table S6).

Further, we explored the transcriptome profile of γδT cells (GSE123649) in duodenal tissue from HC and CeD patients to verify whether pyroptosis and IFN-γ pathway were directly mediated by these cells. DEGs analysis showed that the expression of IFN-γ in γδT cells was significantly increased (fold change = 4.92) in CeD duodenal tissue (Fig. 6D, Additional file 8: Table S7), suggesting that the cells were an important source of IFN-γ, and might partly contribute to the activation of duodenal IFN-γ pathway in CeD patients. On the contrary, the results of PES showed no difference in γδT cells between the CeD and HC groups (Fig. 6D), suggesting that the process of pyroptosis did not occur in γδT cells.

### Induction of duodenal epithelial cells to become “pre-pyroptotic” cells by γδT cells-derived IFN-γ

**Fig. 7:**
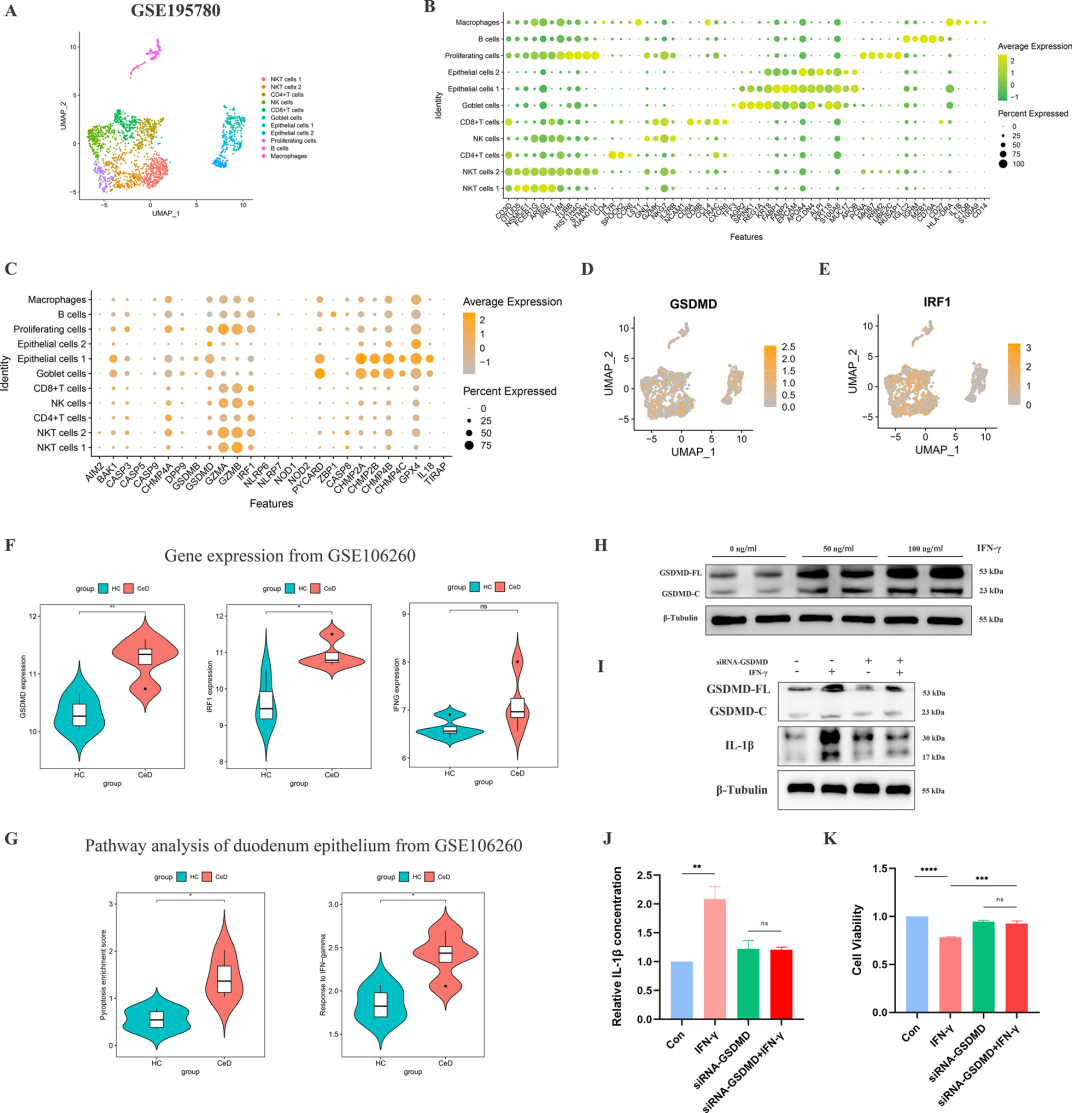
Regulation of IFN-γ/IRF1/GSDMD axis induced epithelia to be in “pre-pyroptotic condition. **A** The UMAP embedding identified 11 kinds of cells in duodenal mucosa from refractory celiac disease (RCD) patients. **B** The dot plot showed the average expression and expressed percentage of the marker genes. **C** The dot plot showed the average expression and expression percentage of pyroptosis-related genes. **D** The feature plot demonstrated the distribution and expression of GSDMD. **E** The feature plot demonstrated the distribution and expression of IRF1. **F** The comparison of GSDMD, IRF1 and IFNG expression level between epithelial cells from CeD patients and HC in dataset GSE106260. **G** The comparison of PES and the response to IFN-γ pathway, and IFNG expression level between epithelial cells from CeD patients and HCs in dataset GSE106260. **H** Western blotting showed GSDMD-FL, GSDMD-C and β-tublin of Caco-2 cell line stimulated by 0 ng/ml, 50 ng/ml and 100 ng/ml IFN-γ, respectively. **I** Western blotting showed GSDMD-FL, GSDMD-C, IL-1β and β-tublin of Caco-2 cell line treated with IFN-γ (100 ng/ml) and/or siRNA-GSDMD. **J** Concentration of IL-1β the supernatant of Caco-2 cell line treated with IFN-γ (100 ng/ml) and/or siRNA-GSDMD was measured by ELISA. **K** Cell viability in Caco-2 cell line treated with IFN-γ (100 ng/ml) and/or siRNA-GSDMD was measured by CCK-8 assay

To further clarify how did IFN-γ induce pyroptosis, on the one hand, we analysed the expression of genes and proteins related to pyroptosis in the duodenum through the Human Protein Atlas (HPA) database (Additional file 2: Fig. S2C) and found that GSDMD, one of the downstream molecules of IFN-γ also an important pyroptosis-mediating protein, was mainly distributed in epithelial cells (Additional file 2: Fig. S2D). To verify whether the expression of GSDMD was still higher in the epithelium of CeD duodenal tissue, we employed a single-cell RNA sequence dataset (GSE195780) from duodenal tissue of patients with refractory CeD (Fig. 7A, B). Expression analysis showed that GSDMD was significantly enriched in a variety of epithelial cells, including absorptive epithelial, goblet, and proliferative cells (Fig. 7C–E), suggesting that epithelial cells were the main targets of the pyroptosis process.

On the other hand, by analysing the transcriptome sequencing of duodenal epithelial tissue (from dataset GSE106260), we found that GSDMD and IRF1 (an IFN-γ-adjusted transcriptional regulator which was involved in apoptosis, immune response, and DNA damage response) were significantly higher in patients with CeD than in normal subjects (Fig. 7F). Meanwhile, the PES in CeD epithelia was significantly higher than in normal counterparts (Fig. 7G). In vitro studies, stimulation of IFN-γ significantly upregulated the expression of both GSDMD full length (GSDMD-FL) and cleaved GSDMD (GSDMD-C) but not the enhancement of cleaved ratio in epithelial Caco-2 cell line (Fig. 7H, Additional file 3: Fig. S3E), while knockdown of GSDMD by siRNA significantly downregulated the expression of GSDMD-FL and GSDMD-C, reduced the actived IL-1β level and the release of IL-1β (Fig. 7I, J, Additional file 3: Fig. S3F), finally improved cell viability (Fig. 7K). In conclusion, the above evidence suggested a possible mechanism in CeD: the overexpression of IFN-γ by γδT cells acted on duodenal epithelial cells and upregulated expression of GSDMD through IRF1, transforming epithelial cells into “pre-pyroptotic” cell which was prone to pyroptosis under external stimulation.

### Gluten is the key factor in increasing γδT cells infiltration, IFN-γ production and triggering the occurrence of epithelial pyroptosis in CeD

**Fig. 8:**
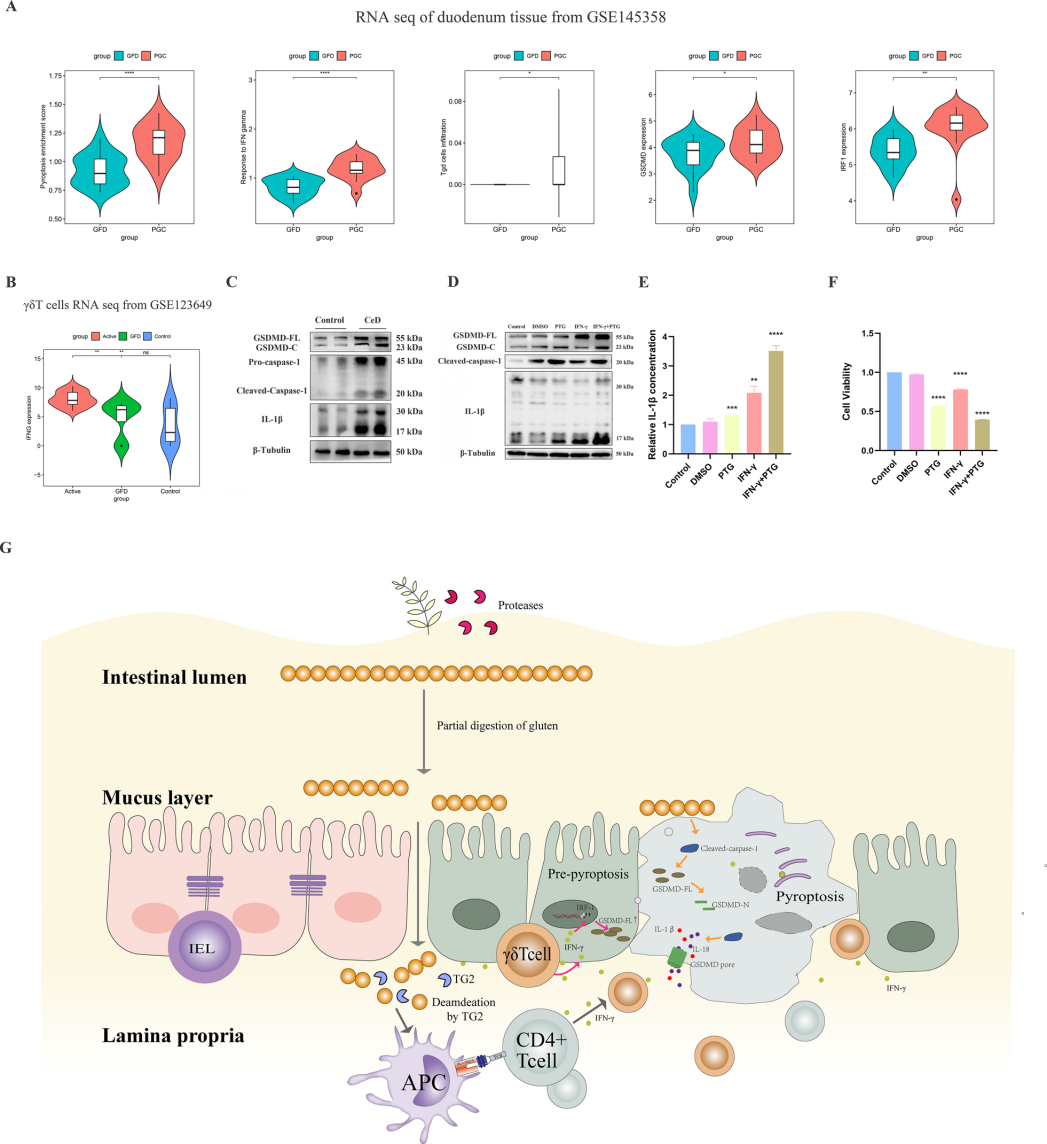
Increasing γδT cells infiltration, IFN-γ response and pyroptosis were triggered by gluten. **A** The comparison of PES, response to IFN-γ pathway, γδT cells infiltration degree, expression level of GSDMD and IRF1 between gluten free diet (GFD) patients and post gluten challenge (PGC) patients in dataset GSE145358. **B** The comparison of γδT cells-derived IFNG expression level among HCs, active CeD patients and GFD patients in dataset GSE123649. **C** Western blotting showed the expression level of GSDMD-FL, GSDMD-C, Pro-caspase-1, Cleaved caspase-1, IL-1β and β-tublin in CeD or HC animal duodenal tissues. **D** Western blotting showed the expression level of GSDMD-FL, GSDMD-C, Cleaved caspase-1, IL-1β and β-tublin in Caco-2 cell line treated with IFN-γ (50 ng/ml) and/or Pepsin-trypsin gliadin (PTG) (200 μg/ml). **E** Concentration of IL-1β in the supernatant of Caco-2 cell treated with IFN-γ (50 ng/ml) and/or Pepsin-trypsin gliadin (PTG) (200 μg/ml) was measured by ELISA. **F** Cell viability in Caco-2 cell treated with IFN-γ (50 ng/ml) and/or Pepsin-trypsin gliadin (PTG) (200 μg/ml) was measured by CCK-8 assay. **G** Hypothetical mechanism of γδT cells mediating pyroptosis in CeD pathogenesis. Patients with a genetic predisposition to CeD could not completely digest gluten.The intermediate products were taken up by antigen-presenting cells and then transferred to the lymphocytes. Upon stimulation, γδT cells produced a large amount of IFN-γ, which upregulate the expression of GSDMD in epithelial cells via IFN-γ/IRF1/GSMDM axis, led epithelial cells to be in a “pre-pyroptotic” condition. Then, epithelia pyroptosis were triggered by PTG/Caspase-1/GSDMD/IL-1β pathway, resulting in a programmed cell death and the release of inflammatory mediators, leding to duodenal villi atrophy and malabsorption of nutrients

Since most studies had demonstrated that gluten is an indispensable factor in the pathogenesis of CeD, we naturally delved into its role to verify whether the functional changes in transcriptional levels were caused by gluten challenge. We investigated a pairing cohort of duodenal transcriptome profiles from 15 CeD patients before and after a gluten diet challenge (GSE145358). A significant upregulation of pyroptosis pathway and IFN-γ response were observed after the gluten diet challenge (Fig. 8A). The expression of GSDMD and IRF-1 were also upregulated in the post-gluten challenge (PGC) group (Fig. 8A). However, no obvious change in the infiltration of γδT cells was noted in CeD patients recieving gluten-free diet (GFD) (Fig. 8A), suggesting that gluten might influence the biological function of γδT cells, but not its quantity. Therefore, dataset GSE123649 was investigated again and found that the expression of IFN-γ in γδT cells was higher in the PGC group compared with the GFD counterpart (Fig. 8B), while no significant difference was observed between gluten-free-diet patients and healthy controls (Fig. 8B), intimating that the upregulation of the response to IFN-γ in CeD patients partly resulted from γδT cells.

Moreover, stronger evidence from CeD animal model confirmed that the GSDMD-FL and cleaved body GSDMD-C levels were higher in the IFN-γ/gliadin group than in the control counterpart, and the increasing cleaved caspase-1 and IL-1β directly verified the occurrence of pyroptosis (Fig. 8C). Co-stimulation of IFN-γ and pepsin-trypsin gliadin (PTG) in Caco-2 cells dramatically promoted the activity of caspase-1 (Fig. 8D), increased the cleaved modification of GSDMD to promote the formation of cell perforation (Fig. 8D), then mediated the release of IL-1β (Fig. 8E) and cell pyroptosis (Fig. 8F).

In summary, our study proposed a possible scientific hypothesis: γδT cells and killer cells in the duodenal tissue of CeD patients increased significantly. Upon stimulation by gluten, γδT cells secreted a large amount of IFN-γ, which then induced epithelial cells to become “pre-pyroptotic” cells. Subsequently, stimulation of intestinal epithelial cells by gluten triggered pyroptosis, resulting in intestinal epithelial atrophy and malabsorption (Fig. 8G).

## Discussion

Current studies illustrated that gluten intolerance was the key factor in the pathogenesis of CeD. However, the mechanism of CeD pathogenesis has not been clarified [33]. This study systematically analysed the differential genes and functional enrichment changes in CeD tissue using scientifically combined multiple microarray and transcriptome sequence data; it further confirmed that γδT cells, a subgroup of T cells, were involved in the pathogenesis of CeD and mediated epithelial pyroptosis via IFN-γ.

Histologically, the duodenum has long intestinal villi, and many microvilli are distributed on the epithelial luminal side, which create a tremendous surface area for efficient absorption of a variety of nutrients [34]. The most significant pathological change in CeD condition is the duodenal villi atrophy, leading to malabsorption, diarrhoea, weight loss, and malnutrition. Although the root cause of duodenal villi atrophy is not clear, repeated mucosal inflammation and programmed epithelial cell death are considered as vital causes [35]. DEGs analysis revealed that downregulated genes in CeD tissue were primarily concentrated on nutrients transport and absorption and changes in metabolic process, confirming that malnutrition in patients with celiac disease was associated with duodenal villi atrophy.

Tissue-resident lymphocytes, including αβT and γδT cells, function as immune monitors and regulators [36]. During the pathogenesis of CeD, the elevated infiltration of γδT cells was observed both in our study and a previous report [5]. However, unlike most T cells, the function and source of γδT cells have not been fully elucidated. Gluten-induced inflammation might trigger not only the change in the number of γδT cells but also their function through the rearrangement of TCR [7]. Our study also revealed an increased infiltration of γδT cells in CeD and the functional changes in IFN-γ expression, which was a pivotal factor in CeD pathogenesis.

Pyroptosis, also known as inflammatory necrosis, is a kind of programmed cell death which is characterised by the continuous expansion of cells and rupturing of the cell membrane, resulting in the release of cell contents such IL-1β, leading to a strong inflammatory response [29]. Since no explicit study has reported the occurrence of pyroptosis in the small intestinal epithelial cells, our study constructively established a relationship between pyroptosis and γδT cells in CeD. Induced by a high level of IFN-γ derived from γδT cells, epithelial cells entered a “pre-pyroptotic” condition, with high expression of GSDMD, which were prone to pyroptosis after stimulation by pepsin-trypsin gliadin or other exogenous toxins. Nonetheless, this hypothesis needs to be further explored employing in vitro and in vivo experiments.

In addition, our study identified 21 hub genes with significant discrepancy expression between CeD and HC patients, including 20 genes that were also distinctly associated with pyroptosis based on WGCNA. Among them, single-cell RNA sequence analysis revealed that IRF1, one of the hub gene higher expressed in CeD epithelial cells, was a transcriptional regulator which regulated the transcription of IFN and IFN-inducible genes and participated in the regulation of many genes expression [37]. In vitro experiments showed that IFN-γ could upregulate the protein level expression of GSDMD in Caco-2 epithelial cell line, while knockdown of GSDMD would lead to effective inhibition of IL-1β release and reduce the occurrence of pyroptosis. Additionally, IFN-γ was highly expressed in γδT cells, which induced a profound transformation in mucosal immune response. Interestingly, IFN-γ was not only regulated by the downstream factor IRF1 but also an upstream element regulator to activate expression of IRF1 [38]..The above evidence indicated an IFN-γ/IRF1/GSDMD signal transduction axis which played a nonnegligible role in pyroptosis.

Gluten is a trigger of pyroptosis, which not only regulates the expression of IFN-γ but also influences the infiltration of γδT cells. High PES was demonstrated in duodenal tissue of CeD patients after gluten challenge, suggesting that pyroptosis might be regulated by gluten. After intake of gluten, the infiltration of γδT cells was increased, while the gluten-free diet markedly rebuilt the expression of IFN-γ in γδT cells. In vitro research on Caco-2 cells further claimed that combined administration of gluten and IFN-γ could activate caspase-1, which cleaved GSDMD-FL into GSDMD-N and caused cell perforations, promoting the release of IL-1β and mediating pyroptosis. Above all, we conceived that gluten promoted the upregulation of IFN-γ expression in γδT cells and changed the quantity of γδT cells, which together resulted in epithelial cells pyroptosis.

Due to no specific symptoms, serological marker, and significant endoscopic and pathological changes in the pathogenesis of CeD, its clinical diagnosis (especially mild or early type) is often missed or mistaken. Based on the LASSO regression model, our findings propose new diagnostic strategies including γδTCR and PES, which have shown considerable distinction in predicting the incidence of CeD in both derivation and validation cohorts.

## Conclusions

In conclusion, by uniting multiple transcriptome profiles, our study constructed an immune cell landscape of CeD, and established a novel association between immunity, inflammation, and programmed cell death. While individuals with the genetic background of CeD were stimulated by a gluten diet, a significant infiltration of γδT cells would be initiated in their duodenal mucosal. By producing a large amount of IFN-γ, γδT cells promoted the expression of GSDMD in epithelium by upregulating IRF1 and triggered epithelial cells to be in a “pre-pyroptotic” state. After gluten intake, epithelial cells underwent pyroptosis, resulting in an irreversible programmed cell death which eventually led to duodenal villi atrophy and malabsorption of nutrients. These conclusions might provide new insight into the cellular and molecular mechanisms, facilitate the clinical diagnosis, and propose novel ideas for the treatment of CeD.

## Supplementary Information

**Additional file 1: Figure S1.** Response to IFN-γ pathway score, immune infiltration analysis of the derivation and validation cohort and co-expression network analysis of the validation cohort. **A** The derivation cohort data before and after removal of batch effect. The batch effect was shown by box chart and PCA analysis. **B** The validation cohort data before and after removal of batch effect. The batch effect was shown by box chart and PCA analysis. **C** The boxplot showed the comparison of IFN-γ response-related genes between CeD and HC tissues in the derivation cohort. **D** The boxplot showed the comparison of response to IFN-γ score between CeD and HC tissues in the derivation cohort. **E** The boxplot showed the comparison of response to IFN-γ score between CeD and HC tissues in the validation cohort. **F** The stacking histogram exhibited the infiltration proportion of all cell types and difference comparison of the infiltration score of all cell types between CeD and HC tissues in the derivation cohort. **G** The stacking histogram exhibited the infiltration proportion of all cell types and difference comparison of the infiltration score of all cell types between CeD and HC tissues in the validation cohort. **H** The correlation heatmap reflected co-expression patterns among immune cell infiltration score, PES, and response to IFN-γ pathway in the validation cohort.

**Additional file 2: Figure S2.** The ridge regression model of the validation cohort and the expression distribution of GSDMD. **A**,** B** The ridge regression model of the validation cohort was constructed using the LASSO regression model. **C**, **D** Distribution of GSDMD RNA and protein expression in normal duodenum tissues according to HPA database.

**Additional file 3: Figure S3.** Dimensionality reduction of single-cell RNA sequence data, cell types annotation and expression distribution of pyroptosis related genes. **A** The UMAP embedding identified 11 kinds of cells without annotation. **B** The heatmap showed the top 10 marker genes of each cell type cluster. **C** The dot plot showed the average expression and the expression percentage of the typical marker genes of each cell type cluster. **D** The feature plot demonstrated the distribution and expression of all pyroptosis- related genes. **E** Western blotting showed the expression level of GSDMD-FL and GSDMD-N in Caco-2 cell line treated with IFN-γ (100 ng/ml) for different time points. **F** RT-qPCR showed the expression level of GSDMD in Caco-2 cell line treated with IFN-γ (100 ng/ml) and/or siRNA-GSDMD.

**Additional file 4: Table S1.** The DEGs analysis between CeD and HC duodenal tissues in derivation cohort. 

**Additional file 5: Table S2–S4.** The GO, KEGG and Reactome analysis of DEGs between CeD and HC duodenal tissues in derivation cohort. 

**Additional file 6: Table S5.** Pyroptosis associated genes identified from the Reactome database, GO database, and previous reports. 

**Additional file 7: Table S6.** Pyroptosis associated genes used in the "pyroptosis enrichment score" model. 

**Additional file 8: Table S7.** The DEGs analysis between CeD and HC duodenal γδT cells.

## Data Availability

The data of this study derived from publicly available datasets, with datasets (GSE72625, GSE112102, GSE164883, GSE131705, GSE134900, GSE146190, GSE125625, GSE191015, GSE123649, GSE106206, GSE195780, GES145358) provided by Gene Expression Omnibus can be found here: GEO, https://www.ncbi.nlm.nih.gov/geo/. Other materials in this study, data process and R codes were descripted in Methods parts and supplementary materials.

## Abbreviations

DAPI: 4′,6-Diamidino2-phenylindole
AUC: Area Under Curve
CeD: Celiac disease
DEGs: Differentially expressed genes
FITC: Fluorescein Isothiocyanate
GEO: Gene Expression Omnibus
GO: Gene Ontology
GSEA: Gene set enrichment analysis
GFD: Gluten-free diet
HC: Healthy control
HPA: Human Protein Atlas
IFN-γ: Interferon-gamma
KEGG: Kyoto Encyclopedia of Genes and Genomes
LASSO: Least absolute shrinkage and selection operator
PTG: Pepsin-trypsin gliadin
PGC: Post-gluten-challenge
PCA: Principal component analysis
PPI: Protein–protein interaction
PES: Pyroptosis enrichment score
PAGs: Pyroptosis-associated genes
ROC: Receiver Operating Characteristic
STRING: Search Tool for the Retrieval of Interacting Genes
scRNAseq: Single cell RNA sequence
ssGSEA: Single sample gene set enrichment analysis
UMAP: Uniform Manifold Approximation and Projection
WGCNA: Weight gene correlation network analysis
Tgd cells: γδT cells
