# Supplementary figures and images for: Transcriptome profile and immune infiltrated landscape revealed a novel role of γδT cells in mediating pyroptosis in celiac disease
Source: J Transl Med. 2023 Jul 24;21:497. doi: 10.1186/s12967-023-04359-1 (PMC10364383; doi:10.1186/s12967-023-04359-1)

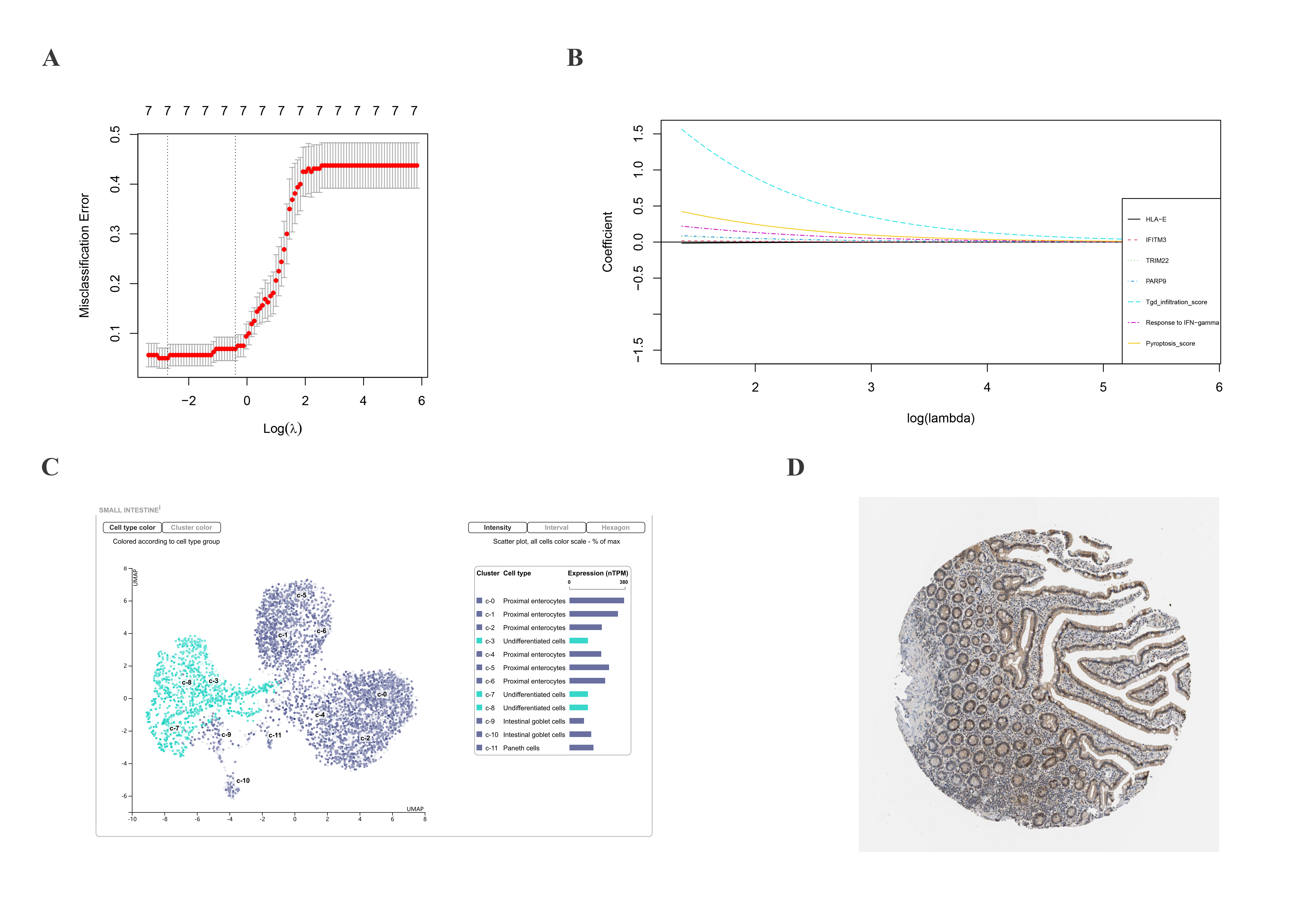

Supplement: Supplementary file 2 — Additional file 2: Figure S2. The ridge regression model of the validation cohort and the expression distribution of GSDMD. A, B The ridge regression model of the validation cohort was constructed using the LASSO regression model. C, D Distribution of GSDMD RNA and protein expression in normal duodenum tissues according to HPA database. [file 12967_2023_4359_MOESM2_ESM.png]
